# Supradiaphragmatic ectopic liver

**DOI:** 10.11604/pamj.2022.41.152.33696

**Published:** 2022-02-21

**Authors:** Savas Deftereos, Maria Kalbazidou

**Affiliations:** 1 Radiology Department, Democritus University of Thrace, Alexandroupolis, Greece

**Keywords:** Supradiaphragmatic ectopic liver, hemidiaphragmatic eventration, computed tomography

## Image in medicine

An 81-year-old man was referred to our hospital for unexplained weight loss (more than 7 kilograms in last 6 months). The complete blood count shows evidence of anaemia. The findings of clinical examination were unremarkable and no other comorbidities or coexisting symptoms was referred. His past medical history includes arterial hypertension. The findings from the contrast enhanced computed tomography of the abdomen depicted a 2.7cm multi-chambered liver cyst compatible with echinococcal cyst, cholelithiasis without signs of inflammation, diverticulosis and no obvious reason for weight-loss. However, a lobulated right subdiaphragmatic liver surface (huge hump like) was noticed. The referred area’s high was 6,2cm and the “base” was 6.7cm. The hump appeared to be continuous with the right hepatic lobe via a wide surface and had similar architecture and vessel continuity with the liver. Supradiaphragmatic ectopic and in generally the occurrence of ectopic liver tissue is rare (incidence: <0.5%). In this anatomical variant, liver tissue extends into the right hemithorax through an opening in the right hemidiaphragm. This differs from a transdiaphragmatic congenital herniation of the liver, in that no apparent defect depicted in the diaphragmatic complex besides the normal hiatus. Furthermore, patient must not have a history of trauma, otherwise traumatic liver herniation through a diaphragmatic tear must be more likely. It is important for radiologists to consider supradiaphragmatic ectopic liver as a differential diagnosis when facing intrathoracic “mass” in continuation with right hemidiaphragm and/or right hemidiaphragmatic eventration. The figure actually includes: contrast enhanced computed tomography: a) multiplanar reconstruction showing the supradiaphragmatic ectopic liver and its vascularity (arrow). Star: echinococcal cyst; b) axial view: showing a mildly enhancing well circumscribed, soft tissue mass in the right hemithorax.

**Figure 1:**
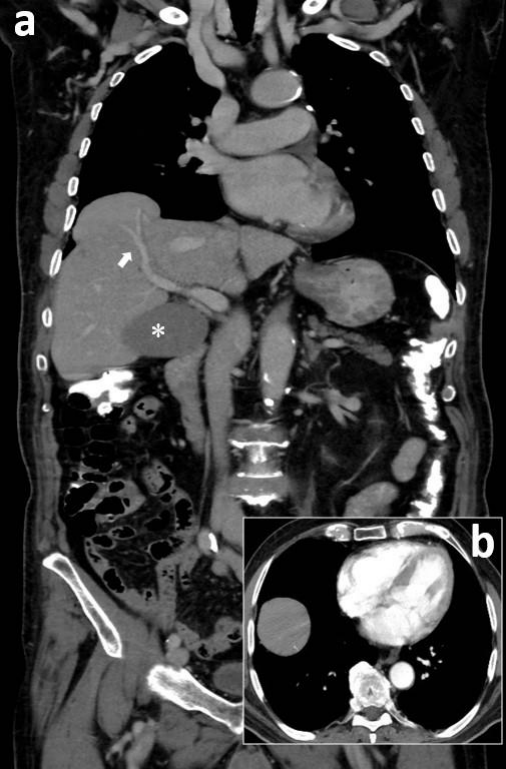
contrast enhanced computed tomography; a) multiplanar reconstruction showing the supradiaphragmatic ectopic liver and its vascularity (arrow); star: echinococcal cyst; b) axial view: showing a mildly enhancing well circumscribed, soft tissue mass in the right hemithorax

